# Modified external fixation technique for treatment of distal radial metaphyseal-diaphyseal junction fractures in pediatric patients

**DOI:** 10.3389/fped.2025.1719011

**Published:** 2025-12-08

**Authors:** Huanye Zhu, Chao Gao, Mengyao Wang, Nan Zhang, Jin Cao

**Affiliations:** 1 Department of Pediatric Orthopedics, Ningbo No. 6 Hospital, Ningbo, Zhengjiang, China; 2 Ningbo Clinical Research Center for Orthopedics, Sports Medicine & Rehabilitation, Ningbo, Zhejiang, China

**Keywords:** pediatric fractures, distal radial metaphyseal-diaphyseal junction, minimally invasive treatment, external fixation, early functional rehabilitation

## Abstract

**Background:**

Distal radius metaphyseal-diaphyseal junction (DRMDJ) fractures in children are often unstable and frequently require surgical intervention. Although various clinical approaches are available, each has its limitations. We introduced a modified external fixation technique for managing pediatric DRMDJ fractures. This study aimed to evaluate the clinical efficacy of this technique.

**Methods:**

Between June 2021 to June 2024, a total of 57 pediatric patients with DRMDJ fractures were reviewed retrospectively. All patients were managed using a modified external fixation technique that incorporated distraction closed reduction and fixation, thereby facilitating early functional recovery without the need for cast immobilization. Postoperative follow-up was conducted at scheduled intervals and included radiographic evaluation (x-rays), assessment of wrist function scores, and recording of patient satisfaction metrics.

**Results:**

Successful closed reduction and application of external fixation were achieved in all 57 pediatric patients. Throughout the follow-up period, no cases of nonunion or fixation failure were observed. The treatment protocol facilitated early functional rehabilitation, as evidenced by a progressive and statistically significant improvement in Cooney scores: from 74.5 ± 10.7 at 3 weeks to 87.5 ± 7.2 at 6 weeks, 93.5 ± 4.5 at 3 months, and 96.8 ± 2.7 by 6 months postoperatively (*p* < 0.05). Postoperative surveys indicated high rates of satisfaction among patients and their families.

**Conclusion:**

The modified external fixation technique for pediatric DRMDJ fractures enables early functional rehabilitation and a quicker return to school activities, while obviating the need for secondary hardware removal surgery. This approach adheres to minimally invasive principles and cosmetic acceptability, positioning it as an effective therapeutic strategy for treating pediatric DRMDJ fractures.

## Introduction

Distal radius fractures represent one of the most common pediatric fractures, typically resulting from falls or trauma (1). While many could be managed conservatively, those with significant displacement or instability often require surgical intervention (2, 3). In recent years, significant advancements have been made in understanding fractures of this region (4–7). Lieber et al. (5) introduced the concept of the distal radial metaphyseal-diaphyseal junction (DRMDJ), identifying fractures in this area as a distinct type of distal radius fractures. Pediatric DRMDJ fractures are characterized by a “back-to-back” cortical deformity, resistance to manual reduction, and inherent instability, frequently necessitating surgical management.

Elastic stable intramedullary nailing (ESIN) is a widely used technique in pediatric orthopedics and has been documented for treating DRMDJ fractures with various nail insertion approaches (8–10). However, a notable limitation of ESIN is its inability to directly reduce fracture fragments, requiring preliminary reduction through leverage or open reduction prior to fixation. Although some scholars (11) have employed plate fixation, this method is frequently declined by parents due to its invasive nature and the requirement for secondary surgery to remove the implants.

In response to these limitations, our institution has adopted an external fixation technique for the closed reduction and stabilization of pediatric DRMDJ fractures. This article details the technical procedure, shares pertinent clinical insights, and provides a preliminary evaluation of its therapeutic efficacy.

## Patients and methods

### Patients

Between June 2021 to June 2024, we retrospectively collected 57 pediatric patients with the DRMDJ fractures, all were treated using a modified external fixation technique. The inclusion criteria: (1) Children diagnosed with DRMDJ fracture through clinical and imaging examinations, (2) Patients with failed manual reduction or unstable fractures requiring surgical intervention, (3) Patients aged 6–14 years old. The exclusion criteria: (1) Involvement of the distal radius epiphysis or articular surface, (2) Pathological fracture, open fracture, old fracture or associated neurovascular injuries, (3) Combined with displaced ulnar fracture requiring surgical treatment, (4) Combined with other systemic diseases that cannot cooperate with treatment. The study was approved by the hospital ethics committee, and informed consent was obtained from the guardians all patients. Written informed consent was obtained from all legal guardians.

### Operational principles of external fixation for pediatric DRMDJ fractures

**Figure 1:**
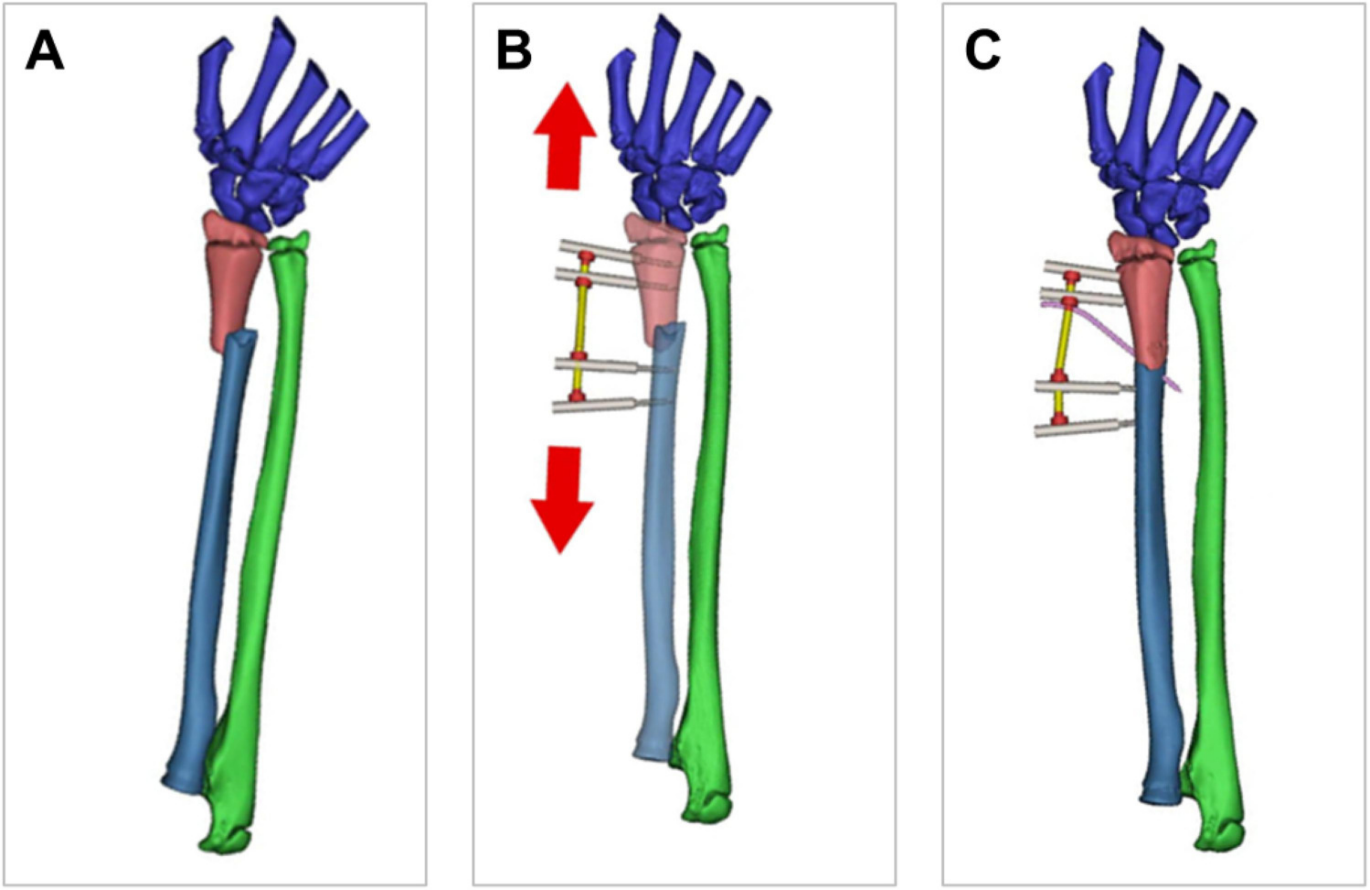
Principles of external fixation technique. **(A)** Anatomical configuration of DRMDJ fractures. **(B)** Distraction-reduction mechanism of external fixation technique. **(C)** Adjunctive K-wire was used to stabilize the fracture ends and prevent rotation.

Successful application of external fixation requires comprehensive knowledge of pediatric distal radius anatomy. The distal radius occupies a subcutaneous position with minimal soft tissue coverage and well-defined surface landmarks. While critical neurovascular structures are situated on the volar aspect, extensor tendons traverse along the dorsal aspect of the radius. This anatomical configuration permits safe external fixation pin placement when the distal radial physis is meticulously avoided (Figure 1A). The technique employs a distraction-reduction mechanism, utilizing controlled distraction through fixation pins to achieve fracture reduction. This approach effectively corrects radial shortening and angular deformities (Figure 1B). However, the bridging configuration of external fixation may result in residual fracture site instability. To address this, adjunctive Kirschner wire (K-wire) stabilization is recommended (Figure 1C), which improves mechanical stability and minimizes micromotion during healing.

### Surgical technique

**Figure 2:**
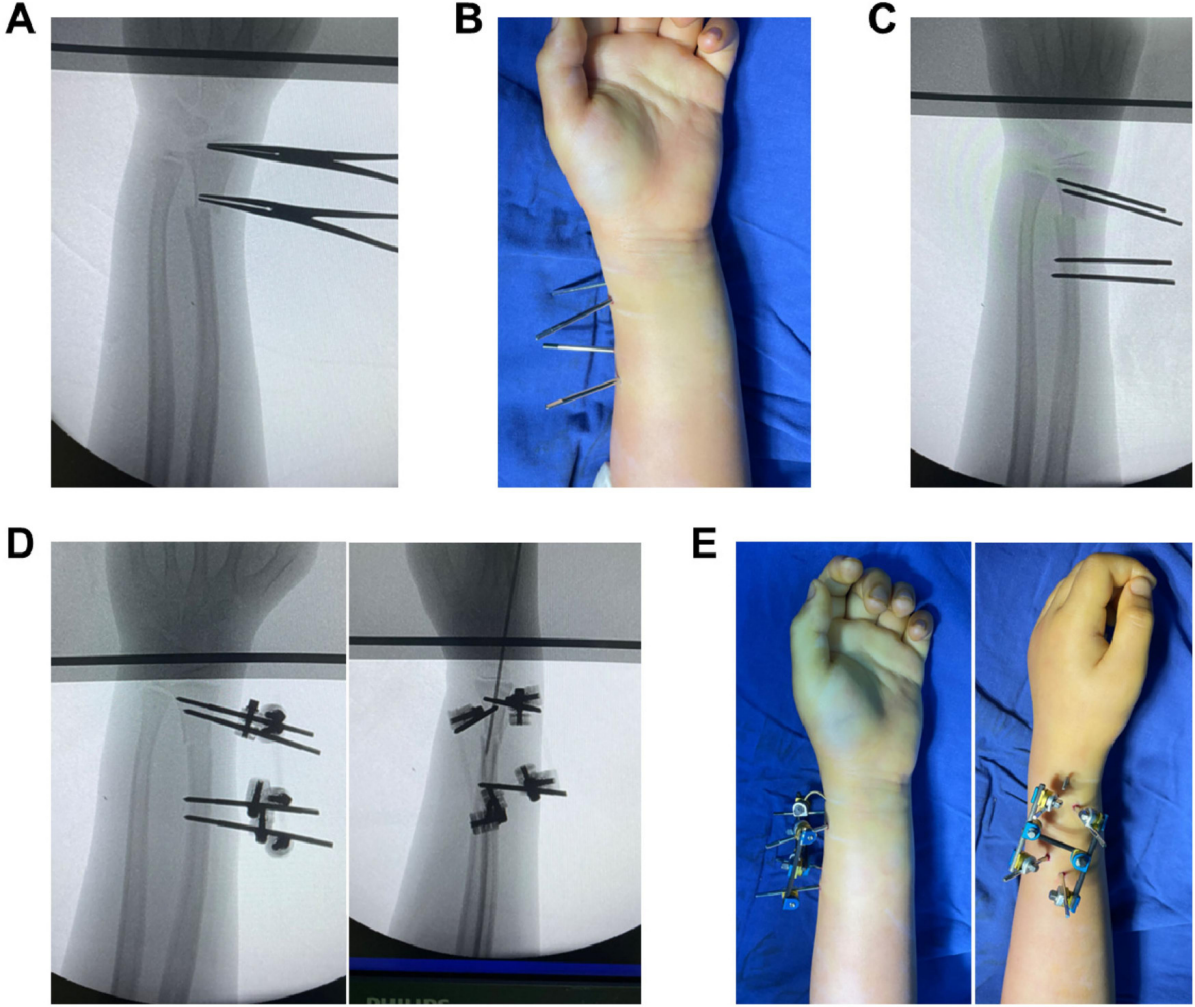
External fixation procedure for pediatric distal radius fractures. **(A)** x-ray localization of fracture and epiphysis. **(B)** Bilateral 2.5 mm pin insertion through stab incision. **(C)** Fixation-assisted closed reduction. **(D)** Supplemental K-wire for stability. **(E)** Minimal scarring for surface.

All surgical procedures were performed by a pediatric orthopedic surgeon with more than 15 years of clinical experience. The surgical procedure was conducted under combined brachial plexus block and intravenous anesthesia, with the patient in the supine position and a upper arm tourniquet applied. Radiation shielding protected the genital and thyroid regions. After standard antiseptic preparation and draping, the affected limb was positioned on a radiolucent table. x-ray identified the fracture site and distal radial epiphysis (Figure 2A), demarcating safe zones for pin placement, we adhered to a strict safety criterion: the needle was inserted no closer than 2.5 mm (one fixation pin length) from the epiphysis, a position confirmed under C-arm guidance. A 0.3 mm stab incision was made at the designated entry point, followed by blunt dissection of subcutaneous soft tissues using a hemostat to establish a channel to the bone surface. Two 2.5 mm fixation pins were then inserted bilaterally through this channel (Figure 2B). A connecting rod of the external fixation (Double Medical Technology Inc., Xiamen, China) was secured to the pins, followed by gradual distraction until achieving satisfactory closed reduction (Figure 2C). After x-ray confirmation of reduction, the fixation was locked. Reduction assessment, we require complete correction of shortening and angulation, and more than two-thirds correction of lateral displacement. To address potential micromotion at the fracture site due to the bridging nature of the external fixation, an Supplemental percutaneous K-wire (Jiangsu Shuangyang Medical Devices Co., Ltd., China) was added to counteract bridging-related micromotion and augment stability (Figure 2D). Final x-ray confirmed proper alignment and apposition of the radial fracture fragments before wound dressing, with minimal scarring for optimal cosmesis (Figure 2E).

### Postoperative management

**Figure 3:**
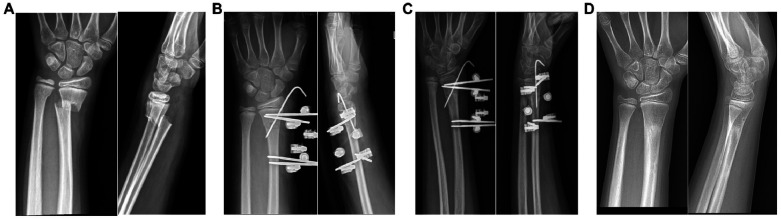
Representative process of the modified external fixation technique. **(A)** Before treatment. **(B–D)** Postoperative follow-up at 3 weeks, 6 weeks and 3 months after surgery.

**Figure 4:**
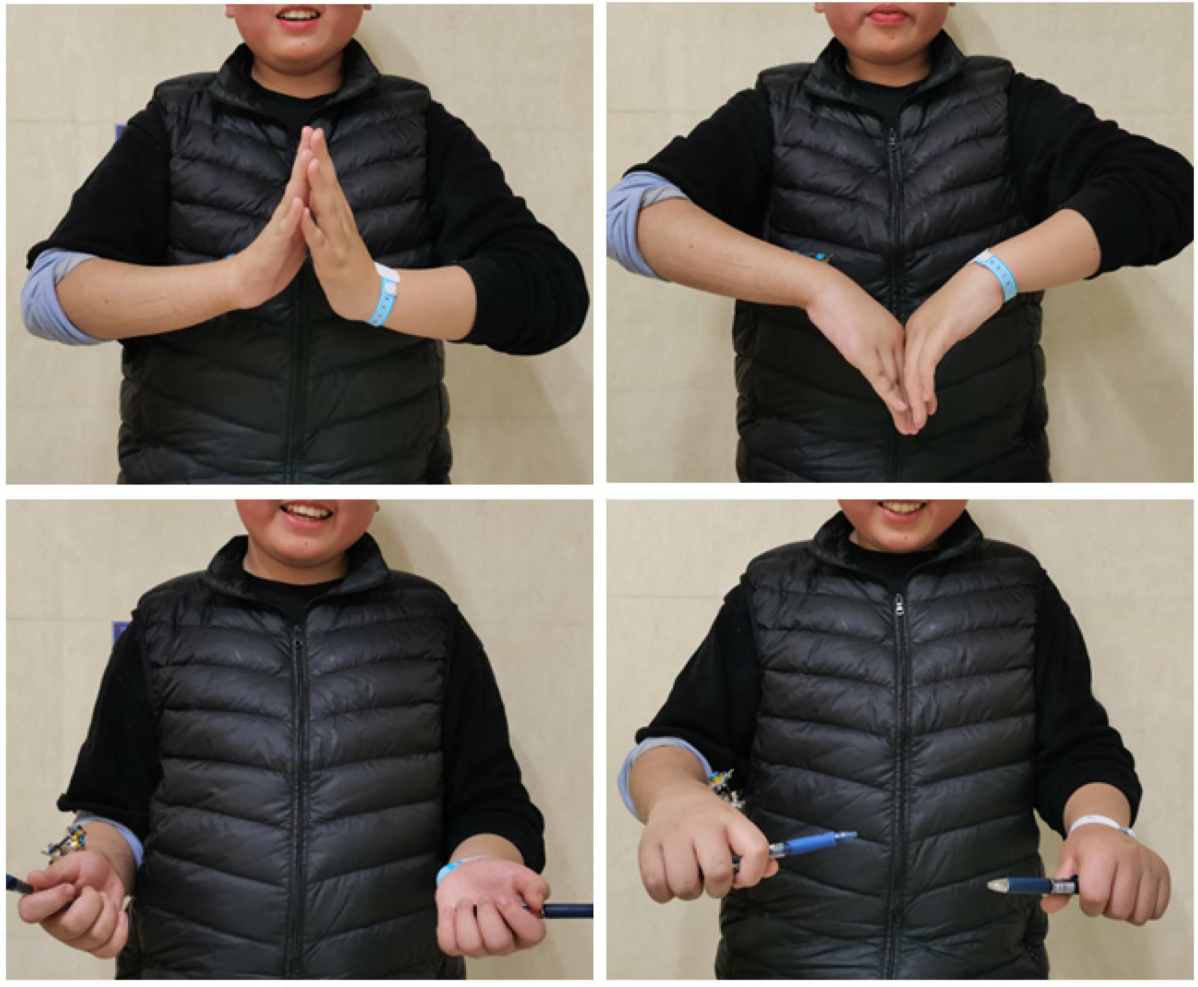
Wrist joint mobility at 3 weeks postoperatively.

Postoperative cast immobilization is unnecessary. Comprehensive patient and family education incorporating verbal counseling and simulated daily activities effectively reduces treatment-related anxiety. Additionally, regular dressing changes and strict pin-site care are required to prevent infection and maintain treatment compliance. Patients may resume daily activities (e.g., writing, utensil handling) and perform non-weight-bearing exercises (e.g., forearm rotation, wrist flexion-extension). Standardized clinical and x-rays follow-ups occurred at 3 weeks, 6 weeks, 3 months, and 6 months after surgery. Fixations were removed upon radiographic confirmation of bridging callus resolved of local tenderness or axial percussion pain at 6 weeks after surgery. We collected patients demographics (age, sex), complications (nerve injury, needle track infection, failure of internal fixation), wrist function scores (3 weeks, 6 weeks, 3 months), and final aesthetic outcomes. A satisfaction questionnaire was administered at the 6-month postoperative visit. Representative cases are illustrated in Figures 3, 4.

### Statistical analysis

Statistical analysis was performed using IBM SPSS software (version 23.0; IBM Corp., Armonk, NY, USA). We used repeated measures ANOVA to analyze the time-dependent trends of the Cooney score, with *p* < 0.05 considered statistically significant.

## Results

As shown in Table 1, our cohort comprised 57 pediatric patients (mean age 9.9 years; range 6–14 years), showing marked male predominance (93% male, *n* = 53). This significant gender imbalance may reflect either greater trauma exposure or severity injury in male patients, potentially predisposing them to complex fractures necessitating surgical management. All cases achieved successful closed reduction with external fixation (mean operative time 58.7 min; range 40–70 min). Complete postoperative follow-up was obtained for all patients, ranging from 6 to 24 months (mean: 12.6 months).

**Table 1:** Basic characteristics of DRMDJ fracture patients.

Index	Data
Age, yr, mean ± SD	9.9 ± 2.4
Sex	
Female, *n* (%)	4 (7.0%)
Male, *n* (%)	53 (93.0%)
Duration of surgery, min, mean ± SD	58.7 ± 7.1
Combined ulna fracture, *n* (%)	53 (93.0%)
Hospital stay, day, mean ± SD	5.5 ± 1.8

Five patients (8.8%) developed pin tract infections, all resolving with daily wound care and oral antibiotic. All infections completely resolved following fixator removal. We serially assessed wrist functional recovery using the Cooney wrist scoring system (12). Although the application of this score in the pediatric field remains to be confirmed, the tool enables clinicians and patients to quantitative track rehabilitation progress. Based on *post-hoc* tests: early rapid recovery phase (3 Weeks to 6 Weeks): the score showed the largest increase (mean difference of 13.0), this suggests the early postoperative period is the stage of fastest functional recovery. mddle steady recovery phase (6 Weeks to 3 Months): the rate of improvement (mean difference of 6.0) slowed compared to the early phase (Table 2).

**Table 2:** Changes in cooney score following fracture surgery over time (*n* = 57).

Time Point	Cooney Score (Mean ± SD)	Repeated Measures ANOVA Result
3 Weeks Post-op	74.5 ± 10.7	F(3, 168) = 85.39, *p* < 0.05
6 Weeks Post-op	87.5 ± 7.2	
3 Months Post-op	93.5 ± 4.5	
6 Months Post-op	96.8 ± 2.7	

A customized patient satisfaction questionnaire was requested from the patient's family at the 6-months follow-up after surgery, which included five questions: 1. Whether the care of the patient after discharge causes trouble to you? 2. Whether there is obvious trouble in the return of the external fixation to campus? 3. Do you accept the removal of external fixation needles in the clinic? 4. Whether the size of the skin scar after the child was satisfactory? 5. Will you recommend this surgical plan, if you have a similar fracture patient? Survey results showed that 45.6% of caregivers encountered difficulties with pin tract maintenance. However, only 28.1% reported psychological concerns regarding school reintegration during external fixation. Outpatient fixation removal received unanimous approval (100%), especially among school-aged children, as it reduced academic disruption. High satisfaction with cosmetic outcomes (96.5%) and strong endorsement rates of this surgical approach were recorded.

## Discussion

DRMDJ in children is anatomically defined as the overlapping area between: (1) a large square constructed using the distal radial and ulnar epiphyseal plates, and (2) a smaller square based on the distal radial epiphyseal plate alone (5). Closed reduction with plaster immobilization suffices for DRMDJ fractures demonstrating simple angulation deformities that achieve satisfactory alignment through manual reduction. However, DRMDJ fractures exhibit higher complete displacement rates and more frequent loss of angular correction post-reduction than standard distal radius fractures, indicating inherent instability (13), surgical intervention becomes necessary (7–9, 14, 15).

Multiple fixation strategies are available for these fractures (11–14, 16, 17). Kirschner wire (K-wire) fixation remains standard for pediatric metaphyseal fractures, DRMDJ fractures present unique challenges: the epiphysis leading to reduced wire purchase, increased risks of fixation failure, and iatrogenic physeal damage during repeated pin insertion (18, 19). ESIN has gained popularity for pediatric long bone diaphyseal fractures owing to its three-point fixation, elastic stability, and minimally invasiveness, with increasingly broad applications (9, 10, 20). Kang et al. (21) reported successful outcomes in 90 pediatric ESIN cases, albeit with 44% requiring limited open reduction. Conventional radial ESIN often proves ineffective for DRMDJ fractures, as the short distal fragment prevents as the short distal fragment prevents three-point fixation. Jiang (8) proposed a new method of retrograde precision shaping ESIN to treat DRMDJ fractures, this technology enhances traditional ESIN techniques by applying multiple plastic deformations for precise fixation, and compared it with retrograde K-wires and anterograde ESIN. The outcomes showed the technique significantly improved the accuracy of realignment, increased the healing rate and accelerated the early rehabilitation process. However, this technique requires high technical proficiency from the surgeon, necessitating precise evaluation of the fracture ends and the plastic endpoint of the ESIN.

External fixation provides unique advantages for DRMDJ fractures owing to the anatomical characteristics of the region: minimal muscular attachments, direct subdermal bone contact, and sparse soft tissue coverage enable precise pin placement. A comparative study by Li (7) demonstrated the superior stability of external fixation over K-wire fixation in DRMDJ fractures. However, existing studies have predominantly focused on fixation efficacy, with limited attention to reduction techniques. In our protocol, C-arm-guided percutaneous pin placement across fracture fragments, combined with gradual distraction using the external fixation, facilitates closed reduction, thereby avoiding the need for open surgery. Furthermore, the rigidity of external fixation allows for early joint mobilization, with functional recovery matching the contralateral limb observed as early as three weeks postoperatively. Compare with traditional K-wire or ESIN techniques, external fixation is characterized by several key advantages. First, gradual distraction and closed reduction achieved through external fixation circumvent the necessity of open surgical reduction. Second, the external fixation can be removed in outpatient settings, avoiding the need for secondary surgery to retrieve internal fixation devices. Third, the rigidity of the external fixator eliminates the need for postoperative cast immobilization, enabling early initiation of wrist functional rehabilitation and facilitating early rehabilitation and a prompt return to school.

However, this study has several limitations. First, the percutaneous pins creates direct pathway for microbial invasion, necessitating rigorous pin tract care to minimize infection risks. Second, cases requiring surgical intervention for concurrent distal ulnar fractures still mandate supplemental fixation with ESIN or K-wires. Third, as a single-center retrospective study without biomechanical evaluation, these findings—despite demonstrating favorable outcomes in 57 cases over three years—require validation through multicenter prospective studies. Fourthly, our study lacks comparison with traditional K-wire and ESIN techniques, we hope to supplement this in future research.

External fixation provides a minimally invasive solution for pediatric DRMDJ fractures by enabling closed reduction, offering robust stabilization, accelerated functional recovery, and timely reintegration into academic activities. The avoidance of secondary surgery for hardware removal aligns with aesthetic and minimally invasive principles. The elevated risk of pin tract infections underscores the necessity for meticulous postoperative care. This technique represents a viable therapeutic option for pediatric DRMDJ fractures, pending further high-level evidence to strengthen clinical recommendations.

## Data Availability

The raw data supporting the conclusions of this article will be made available by the authors, without undue reservation.
